# Existential Well-Being Mediates the Relationship Between Religious Well-Being and Mental Health as Well as Quality of Life in Polish Chronic Pain Patients: Longitudinal Pilot Study

**DOI:** 10.1007/s10943-025-02531-z

**Published:** 2026-01-03

**Authors:** Marcin Wnuk, Maciej Klimasiński

**Affiliations:** 1 Department of Work and Organizational Psychology, Faculty of Psychology and Cognitive Sciences, Adam Mickiewicz University in Poznań, Szamarzewskiego 89/AB, 60-568 Poznań, Poland; 2 Department of Chronic Pain Management in Palliative Care Hospice in Poznan, University of Medical Sciences Poznan, Poznan, Poland

**Keywords:** Chronic pain, Religious well-being, Existential well-being, Spiritual well-being, subjective well-being, Depression, Anxiety, Mental health, Quality of life

## Abstract

Chronic pain is a common medical challenge in Poland. Its impact is particularly significant because it affects all dimensions of patients’ lives. Interestingly, the psychological and spiritual dimensions of quality of life appear to be closely intertwined. There is a scarcity of research explaining the beneficial or detrimental roles of religious coping on well-being in chronic pain patients. However, research on other populations indicates the importance of the existential factor within the two-dimensional spiritual well-being theory. We hypothesize that religious well-being is indirectly linked to mental health and quality of life through its role in facilitating meaning and purpose in life. We recruited 58 non-cancer chronic pain patients at the Pain Management Clinic of the University Clinical Hospital in Poznań, Poland, and examined them in two waves with a three-month interval between February and July 2025. The statistical analysis indicated that religious well-being significantly reduces depression and anxiety and increases quality of life, indirectly through improving existential well-being. We conclude that while belief in God may offer comfort, the psychological benefits of religiosity are conditional and depend on whether it fosters adaptive meaning-making. Purpose and hope may or may not be rooted in religious frameworks. These findings underscore the need for implementing interventions for patients with chronic pain in Poland, which focus on finding meaning and purpose—such as meaning-centered therapy or spiritually oriented conversations in clinical settings.

## Introduction

### Prevalence, Impact, and Holistic Approach to Chronic Pain

Chronic pain is a major medical and social challenge in Poland. In European adult populations, its lifetime prevalence ranges from 12.7 to 33.7% (Rometsch et al., 2025). The likelihood increases with age: In Poland, 42% of adults aged 65 years and over report chronic pain, compared with 35.2% of those aged 55–59 (Kozak et al., 2017). Severe chronic pain affects the quality of life (QOL): physical, psychological, social, and spiritual dimensions of life (Anselmo et al., 2024). Because pain intensity correlates negatively with self-efficacy and positively with pain centrality, depression, and anxiety (Sucher 2020)—biopsychosocial approaches are widely used (Turk & Monarch, 2002). Besides typical psychotherapeutic methods, like cognitive behavioral therapy (Chen et al., 2023), interventions directed at the spiritual dimension take place: mindfulness-based programs (Pagnini et al., 2015), yoga (Metri et al., 2023), physician-led relationship-building activities (Klimasiński et al., 2024), and twelve-step group programs (Bedford, 2025).

### Religion/Spirituality, Mental Health, and Quality of Life

A meta-analysis of 48 longitudinal studies determined the positive longitudinal effect of engagement in religion or spirituality on mental health (Garssen et al., 2020). According to Zarzycka et al. (2019), religion may protect against anxiety because it enhances hope. According to Koenig et al. (1998), associations between religious coping and mental health are at least as strong, if not stronger, than those observed with nonreligious coping. Faith enables some individuals to perceive negative events from, what they believe to be, God’s perspective. In Christianity, it is believed that Jesus, through his death on the cross in the ultimate manifestation of love to humanity, transformed the meaning of suffering. In this and similar ways, religions demonstrate to believers how to effectively cope with adversity and where to find strength in their daily struggles. This concept corresponds to the vertical factor (religious well-being, RWB) included in the two-dimensional spiritual well-being theory. The horizontal factor, on the other hand, constitutes existential well-being (EWB) and refers to a sense of meaning and satisfaction with life without any reference to God (Moberg, 1974; Surzykiewicz et al., 2025).

Previous research has emphasized the pivotal role of spirituality in the lives of patients experiencing chronic pain (Andersen et al., 2020; Braun et al., 2022; Ganasegeran et al., 2020; Lago-Rizzardi et al., 2015). Many individuals do use religiosity to cope with their chronic pain (Jones et al., 2008; Rippentrop, 2005). However, findings on the relationship between religiosity and well-being among chronic pain patients are mixed. Bush et al. (1999) found that among a sample of patients from a pain management clinic, positive religious coping predicted positive psychological outcomes. Similarly, Basiński et al. (2013) examined Polish chronic pancreatitis patients after neurolytic celiac plexus block and found that those reporting higher religiosity or church attendance had significantly better QOL. In contrast, in a sample of Swedish patients with chronic pain, the use of prayer as a coping method was correlated positively with depression and anxiety (Andersson, 2008). There is still a lack of research on the mechanisms underlying the relationship between religiosity and the quality of life as well as the mental health of chronic pain patients (Rippentrop, 2005). Earlier studies have reported that spiritual well-being can be a favorable factor for mental health, protecting against stress, anxiety, and depression in samples other than those with chronic pain (Behmanesh et al., 2025; Khanipour-Kencha et al., 2024; Liu et al., 2023; Parviniannasab et al., 2022; Vakili Sadeghi et al., 2025). For example, in oncology patients, both religious and existential well-being were negatively related to depressive and anxiety symptoms (Vakili Sadeghi et al., 2025). Additionally, among nursing students, a positive direct effect of spiritual well-being on mental health was observed, as well as an indirect effect through psychological capital (Parviniannasab et al., 2022).

This study attempts to examine the role of religion as a meaning-oriented system, which can help individuals derive meaning and purpose in life (Park, 2005; Silberman, 2005). Meaning and purpose, in turn, can lead to better quality of life and improved mental health (Boreham & Schutte, 2023; Park et al., 2018; Wilski et al., 2024; Wnuk, 2022). Some studies have indicated an indirect relationship between religiosity and well-being and mental health, emphasizing the mediating role of finding meaning and purpose in life (Krok, 2015; Wnuk, 2024). For example, in Wnuk’s study (2024), positive religious coping facilitated finding a meaningful and purposeful life, which in turn favors positive affect and inhibits negative affect and feelings of hopelessness. Also, using negative religious coping led indirectly, through finding meaning, to extend positive and inhibit negative emotions and feelings of hopelessness (Wnuk, 2024). In the same vein, Krok (2015) observed that negative religious coping disrupts meaning in life, which in turn positively predicts lower psychological well-being. However, these studies did not focus specifically on chronic pain patients.

### Aim of the Study

As shown above, spiritual well-being is an important goal in the care of chronic pain patients. However, religious interventions are not the way to achieve this goal, because faith cannot be prescribed to anyone, especially since some forms of religious coping yield negative outcomes (Krok, 2015; Park et al., 2018; Wnuk, 2024). Existential well-being, however, appears to enhance quality of life and mental health since it equals to finding meaning and purpose regardless of religious well-being. For instance, Zarzycka et al. (2024) report a positive association between religious well-being and existential well-being, as well as the absence of statistically significant relationships between religious well-being and psychological well-being, depression, and both positive and negative mood. Additionally, they find relevant associations between existential well-being and psychological well-being, depression, and mood. Following these findings, life meaningfulness and achieving existential well-being appear to be the factors that mediate the relationship between religious well-being and positive psychological outcomes. It can mean that existential well-being is a necessary factor in improving the quality of life and limitation of symptoms of depression and anxiety in chronic pain patients who experience religious well-being. Based on the above, we hypothesize that in a sample of Polish patients with chronic pain, RWB at T1 will be related to an increase in EWB at T2 (Hypothesis 1), and that EWB at T2 will positively predict quality of life and mental health at T2 (Hypothesis 2). Additionally, we expect that the relationship between RWB at T1 and quality of life and mental health at T2 will be indirect, through increased EWB at T2 (Hypothesis 3).

In our study, we use the following definitions: Religious well-being refers to the positive relationship with God, a feeling of safety resulting from a deep bond with God, the conviction about being loved and cared for by God, and fulfillment experienced during communion with God (Paloutzian et al., 2021). Existential well-being refers to a sense of meaning and purpose, fulfillment in life, and hope for the future (Paloutzian et al., 2021). Mental health was examined using measures of depression and anxiety, which are commonly used indicators in most studies (Halladay et al., 2024). Quality of life was assessed as a subjective evaluation of life and its quality from the participants’ perspective. Following Koenig and Carey (2025), we verified the potential contamination of the Spiritual Well-Being Scale (SWBS; Paloutzian et al., 2021) by examining its items as potential indicators of mental health. The analysis of the content of items of this measure indicated a lack of this problem. An additional argument in support of this conclusion was that the religious well-being factor was not directly correlated with mental health indicators, and correlations between existential well-being and mental health indicators were at most moderate.

## Method

### Participants

In this study, patients were recruited from the Pain Management Clinic of the University Clinical Hospital in Poznań, Poland. The following inclusion criteria were applied: age ≥ 18 years; pain persisting for ≥ 3 months; pain intensity ≥ 4 on the Numerical Rating Scale (NRS). The patients completed the questionnaires listed below via Google Forms at two time points: February–May 2025 (T1) and June–July 2025 (T2), with a three-month interval.

The study protocol was approved by the Bioethics Committee of the Poznań University of Medical Sciences (approval no. 212/25, dated May 8, 2025). Every participant was informed both verbally and in writing about the study and provided written informed consent regarding research participation.

### Measures

#### Spiritual Well-Being

The Polish version (Zarzycka et al., 2024) of the SWBS (Paloutzian et al., 2021) was applied to examine religious well-being and existential well-being as a two-factor construct, comprising spiritual well-being. The religious well-being dimension consists of 6 items, and the existential well-being dimension consists of 5 items. Items are scored on a 6-point Likert scale (1 = strongly disagree, 6 = strongly agree).

#### Anxiety

Anxiety was tested by the Polish adaptation (Basińska & Kwissa-Gajewska, 2023) of the Generalized Anxiety Disorder Scale (GAD-7; Spitzer et al., 2006). One-factor structure with 7 indicators of this measure was confirmed (Basińska & Kwissa-Gajewska, 2023; Spitzer et al., 2006). Responses are rated on a 4-point Likert scale (0 = not at all, 3 = nearly every day). Higher scores indicate greater severity of anxiety.

#### Depression

The Polish version (Ślusarska et al., 2019) of the Patient Health Questionnaire-9 (PHQ-9) (Kroenke et al., 2001) was used to measure depression. It is a unidimensional scale of 9 items rated on a 4-point Likert scale (0 = not at all, 3 = nearly every day). Higher scores indicate more severe depression.

#### Quality of Life

A single quality-of-life item from the WHOQOL-BREF (Hawthorne et al., 2006), rated on a 5-point Likert scale (1 = very low, 5 = very high), was used to examine quality of life.

## Results

### Statistical Analysis and Preliminary Results

A total of 58 patients were recruited. Sociodemographic data are presented in Table 1. The most common diagnoses were osteoarthritis/intervertebral disk disease. In Table 2, descriptive statistics are presented. As shown in Table 2, the reliability of the tools used in the research was satisfactory, with a Cronbach’s alpha coefficient above 0.8 for all measures.

**Table 1:** Sociodemographic variables for patients with chronic pain (*N* = 58)

Variable	*N*	Percentage or mean
Sex		
Female	37	63.8%
Male	21	36.2%
Age		56.7
Household size		2.1
Education level		
Primary	6	10.3%
Vocational	27	46.6%
High school	13	22.4%
Post-secondary	12	20.7%
Employment status		
Employed	13	22.4%
Retired	35	60.3%
Student	6	10.4%
Unemployed	4	6.9%
Marital status		
Partnered/married	27	46.6%
Single	9	15.6%
Widowed	9	15.6%
Divorced	13	22.2%

**Table 2:** Descriptive statistics for patients with chronic pain (*N* = 58)

Variable	Mean		Standard deviation		Skewness		Kurtosis		Alpha Cronbach coefficient	
	T1	T2	T1	T2	T1	T2	T1	T2	T1	T2
Religious well-being	21.20	21.53	10.89	10.89	−.09	−.14	−1.43	−1.48	.96	.97
Existential well-being	17.82	18.60	6.23	6.51	−.21	−.30	−.52	−.51	.87	.88
Quality of life	3	2.98	1.04	1.11	−.48	−.20	−.36	−.62	–	–
Anxiety	22.79	21.72	6.56	8.08	−.22	−.10	−.52	−.97	.89	.94
Depression	14.19	14.31	5.64	6.20	−.27	.22	−.03	−.83	.83	.86

First, the Pearson correlation coefficient (*r*) values were computed (see Table 3). RWB at T1 was positively associated with EWB at T2 and was unrelated to depressive and anxiety symptoms as well as subjective QOL in T2. EWB in T2 was negatively associated with depression and anxiety in T2 and positively linked to QOL in T2.

**Table 3:** Values of r-Pearson correlation coefficients between research variables for patients with chronic pain (*N* = 58)

	1	2	3	4	5	6	7		
1. Religious well-being (T1)									
2. Existential well-being (T2)	.44**								
3. Quality of life (T2)	.07	.54**							
4. Anxiety (T2)	−.09	−.52**	−.57**						
5. Depression (T2)	−.12	−.58**	−.62**	.79**					
6. Quality of life: covariate (T1)	.20	.54**	.75**	-.48**	−.60**				
7. Anxiety: covariate (T1)	−.09	−.37**	−.32**	.52**	.58**	−.36**			
8. Depression: covariate (T1)	−.17	−.49**	−.38**	.56**	.74**	−.46**	.72**		

^*^p < .05., **p < .01

**Fig. 1:**
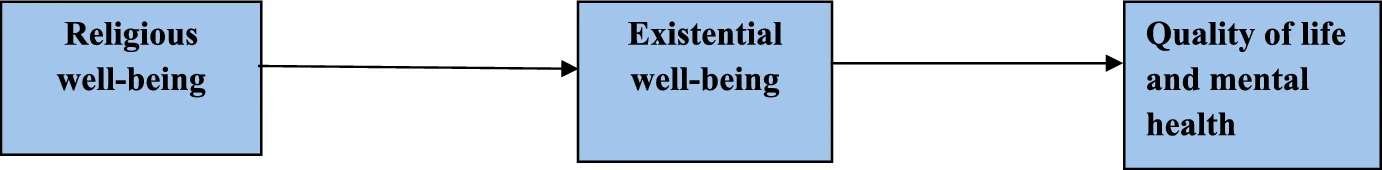
Theoretical model

Next, the research model presented in Fig. 1 was verified in SPSS using Model 4 of the PROCESS macro with the bootstrapping method, employing 5000 resamples (Hayes, 2013). The bootstrapping method is a useful solution in linear regression models (Eck, 2018), especially fruitful in small samples. It allows for estimating confidence intervals by resampling the existing data with replacement, avoiding making assumptions about the data distribution. Also, it helps estimate the reliability of an estimate without making assumptions regarding the original population, providing more accurate internal validity estimates.

In this study, three simple models were tested. Each of them contained an independent variable (RWB in T1), a mediating variable (EWB in T2), one dependent variable (QOL, depression, or anxiety in T2), and one covariate (QOL, depression, or anxiety in T1).

### Hypotheses Testing

Values of standardized total, direct, and indirect effects are shown in Table 4.

**Table 4:** Results of standardized total, direct, and indirect effects for 95% interval (*N* = 58)

Pathway	Value of effect	t	*p*	LLCI	ULCI
Total effects					
Religious well-being: quality of life	−.01	−.76	.4462	−.0256	.0114
Religious well-being: depression	.01	.09	.9280	−.0991	.1085
Religious well-being: anxiety	−.02	−.18	.8559	−.1865	.1554
Direct effects					
Religious well-being: existential well-being	.37	3.42	.0012	.0918	.3502
Existential well-being: quality of life	.27	2.46	.0171	.0086	.0842
Existential well-being: depression	−.34	−3.24	0020	−.5251	-.1240
Existential well-being: anxiety	−.45	−3.65	.0006	−.8766	-2560
Religious well-being: quality of life	−.02	−1.73	.0889	−.0361	.0026
Religious well-being: depression	.08	1.45	.1528	−.0292	.1821
Religious well-being: anxiety	.12	1.45	.1528	−.0478	.2975
Indirect effects					
Religious well-being: existential well-being: quality of life	.09			.0107	.2626
Religious well-being: existential well-being: depression	−.13			−.2443	−.0401
Religious well-being: existential well-being: anxiety	−.19			−.3400	−.0640

LLCI = 95% confidence interval (low); ULCI = 95% confidence interval (high)

Hypothesis 1 connecting RWB in T1 with an increase in EWB in T2 was supported. A positive, significant direct effect of RWB in T1 on EWB in T2 was found. Also, hypothesis 2 about the positive relationship between EWB in T2 and QOL in T2, as well as the negative relationships between EWB in T2 and depression and anxiety in T2, was confirmed. Noted positive direct effect of EWB in T1 on QOL in T2, and negative direct effects of EWB in T2 on depression and anxiety in T2. In accordance with hypothesis 3 regarding EWB in T2 as a mediator between RWB in T1 and QOL and mental health in T2, all examined indirect effects were statistically significant. Noted statistically significant indirect effect of RWB in T1 and QOL in T2, through EWB in T2, the same as the indirect effect of RWB in T1 and depression and anxiety in T2, via EWB in T2. The direct effect of RWB in T1 on QOL in T2 was not statistically significant, the same as the direct effects of RWB in T1 on depression and anxiety in T2.

## Discussion

This study examined the associations between religious well-being, existential well-being, and indicators of quality of life and mental health in patients with chronic pain. The findings support the hypothesis that the beneficial effects of RWB on depression, anxiety, and subjective quality of life are indirect, operating primarily through EWB. In other words, it is not the satisfaction with the bond with God per se that translates into better psychological functioning, but rather the sense of meaning, purpose, and hope that such a relationship can foster. Our findings align with previous studies emphasizing meaning in life as the key mechanism linking religiosity to well-being. Zarzycka et al. (2024) demonstrated that although RWB was positively associated with EWB, it showed no direct relationship with mental health or psychological well-being. In contrast, EWB was negatively associated with depressive and anxiety symptoms and positively related to quality of life. Wnuk and Marcinkowski (2014) showed that hope and meaning in life mediated the relationship between spiritual experiences and subjective well-being indicators. Similarly, Krok (2015) found that religious coping can improve well-being only indirectly through meaning. More recent Polish studies have confirmed that spiritual experiences, forgiveness, hope, and gratitude can strengthen life satisfaction by enhancing meaning in life (Wnuk, 2022; Wnuk et al., 2024). Ferreira-Valente et al. (2022) looked specifically at religiousness and spirituality in chronic pain patients. Their analysis involved 20 experimental and observational studies. They concluded that higher levels of spiritual well-being translated into better physical functioning. Notably, no significant association was found between religiousness and physical functioning.

The absence of direct links between religious well-being and quality of life, as well as anxiety and depression, is theoretically meaningful. It suggests that religiosity alone does not guarantee psychological benefits unless it enhances existential meaning. This interpretation helps explain Andersson’s (2008) finding that frequent prayer among chronic pain patients was associated with higher anxiety and depression. Religious practices may become ineffective—or even distressing—if they do not foster hope, coherence, or purpose. Thus, positive religious effects are conditional, depending on whether faith functions as a source of existential meaning rather than merely ritual engagement.

Our findings also imply that additional mediating factors, other than EWB, not directly measured in our research, may play a role in the RWB–QOL and mental health relationships. For example, moral and interpersonal factors may be involved. Wnuk and Charzyńska (2023) showed that among members of Sexaholics Anonymous in Poland, religious faith facilitated a sense of meaning indirectly via forgiveness. In their model, feeling forgiven by God (and subsequently forgiving others) served as a pathway through which religiosity led to greater perceived meaning in life. This suggests that acts of forgiveness and reconciliation—grounded in one’s religious framework—can bolster the meaning-making process. Our study did not explicitly account for forgiveness or similar spiritually rooted acts, yet the positive associations we observed hint that such factors might be implicitly at work.

These results align with theories that frame religion as a meaning-oriented system (Park, 2005; Silberman, 2005). Religious frameworks may provide interpretive resources that help individuals make sense of suffering and find purpose. This is in line with classic theories (e.g., Frankl’s existential theory; Frankl, 1997) that highlight meaning as fundamental to psychological resilience. The present results empirically support these views. To our knowledge, this is the first study in Poland to examine this mediational model among patients with chronic pain, unlike other Polish studies, which focused on students, healthy adults, or cancer patients (Krok, 2015; Wnuk, 2022; Wnuk & Marcinkowski, 2014; Zarzycka et al., 2024).

These findings should be interpreted in light of their cultural context. Much of the research we cite, as well as our own sample, comes from Poland—a country characterized by a strong Catholic tradition and high levels of religious affiliation, alongside a sizable group of individuals whose religious identification is more nominal than practiced. While the majority of adults continue to declare themselves believers, this proportion has declined over recent decades—from 94% in 1992 to 84% in 2022 (CBOS, 2022). The proportion of weekly churchgoers in Poland has fallen as well—from around 50% in the late 1990s to 36–37% in 2021–2023 (CBOS, 2024). While the Polish Catholic context provides fertile ground for research on the relationship between RWB, SWB, meaning, and psychological well-being, similar mechanisms have been observed elsewhere, particularly where religion operates as a meaning-oriented system (Park, 2005). Thus, our study’s implications likely extend beyond Poland. Culture may shape the expressions of religiosity and the specific content of meaning derived, but the fundamental principle—that existential meaning is the key mediator of religious benefits—appears to hold across diverse contexts.

These findings carry several practical implications for healthcare professionals working with patients experiencing chronic pain. Physicians, psychologists, and chaplains can play a crucial role in supporting patients in reinterpreting their suffering in terms of meaning, purpose, and hope, rather than focusing solely on symptom control. Interventions that strengthen existential well-being—such as meaning-centered therapy, logotherapy, or spiritually oriented conversations in clinical settings—may be particularly beneficial. Importantly, practitioners should differentiate between positive and negative religious coping: The former (e.g., trusting in a benevolent higher power, finding purpose in adversity) tends to enhance well-being, whereas the latter (e.g., spiritual struggle, feelings of abandonment) may exacerbate distress and should be addressed sensitively. Finally, these results suggest that assessing both religiosity and meaning in life may provide a more complete picture of patients’ spiritual and psychological resources, allowing for more targeted and effective supportive care.

### Limitations and Future Research

This study has several limitations that should be acknowledged. First, the sample size was relatively small (*n* = 58), which limits the statistical power and the generalizability of the findings to the broader population of patients with chronic pain in Poland and beyond. Second, the study relied on a single-center sample recruited from one Pain Management Clinic, which may not be representative of patients treated in other settings or regions. Third, although the mediation model fits the data, the temporal sequence of religious well-being, existential well-being, and psychological outcomes was limited to only two time points. Fourth, all variables were measured through self-report questionnaires, which may introduce response bias, including social desirability or recall bias. The absence of objective indicators (e.g., biomarkers of stress, clinician-rated mental health assessments) limits the depth of conclusions. Finally, the study did not control for several potential confounding variables such as pain duration, intensity fluctuations over time, socioeconomic status, or concurrent psychological treatments, which could have influenced the observed relationships.

Future studies should address these limitations by using larger and more diverse samples, incorporating objective outcome measures, and exploring other moderators. Employing longitudinal or intervention designs could determine whether enhancing existential well-being causally improves psychological functioning and quality of life in chronic pain populations. Cross-cultural comparisons, for example, between Poland and more secular societies, could clarify the role of cultural context in religious meaning-making. It would also be valuable to examine different styles of religious coping, distinguishing adaptive from maladaptive patterns, and to integrate this model with other psychological frameworks, such as resilience, self-efficacy, or pain centrality models. Finally, future research should investigate which specific facets of meaning—such as hope, forgiveness, or community belonging—are most relevant across cultural settings.

## Conclusion

Chronic pain poses a major medical and social challenge in Poland. Our findings indicate that the beneficial associations of religiosity on quality of life and mental health in chronic pain patients are indirect, operating primarily through existential well-being. While belief in a higher power may offer comfort, the psychological benefits of religiosity are conditional, depending on whether it fosters adaptive meaning-making. Satisfaction with the bond with God alone is insufficient; the key resource is an inner sense of meaning, purpose, and hope, which may or may not be rooted in religious frameworks. These results underscore the importance of integrating the religious and existential dimensions, which are distinct manifestations of spiritual well-being, into patient care. They suggest that interventions aimed at strengthening meaning in life may enhance well-being in this population.
